# Lipopolysaccharide-Induced Systemic Inflammation in the Neonatal Period Increases Microglial Density and Oxidative Stress in the Cerebellum of Adult Rats

**DOI:** 10.3389/fncel.2020.00142

**Published:** 2020-06-03

**Authors:** Jaime Moreira Pires, Maira Licia Foresti, Clivandir Severino Silva, Débora Bandeira Rêgo, Michele Longoni Calió, Amanda Cristina Mosini, Thabatta Karollynne Estevam Nakamura, Ana Teresa F. Leslie, Luiz Eugênio Mello

**Affiliations:** ^1^Physiology Department, Universidade Federal de São Paulo, São Paulo, Brazil; ^2^Instituto D’Or de Pesquisa e Ensino, Rio de Janeiro, Brazil; ^3^Associação Brasileira de Epilepsia, São Paulo, Brazil; ^4^Biochemistry Department, Universidade Federal de São Paulo, São Paulo, Brazil; ^5^Pediatry Department, Universidade Federal de São Paulo, São Paulo, Brazil

**Keywords:** lipopolysaccharide, neuroinflammation, Purkinje cell, oxidative stress, neonatal

## Abstract

Inflammatory processes occurring in the perinatal period may affect different brain regions, resulting in neurologic sequelae. Injection of lipopolysaccharide (LPS) at different neurodevelopmental stages produces long-term consequences in several brain structures, but there is scarce evidence regarding alterations in the cerebellum. The aim of this study was to evaluate the long-term consequences on the cerebellum of a systemic inflammatory process induced by neonatal LPS injection. For this, neonatal rats were randomly assigned to three different groups: naïve, sham, and LPS. Saline (sham group) or LPS solution (1 mg/kg) was intraperitoneally injected on alternate postnatal days (PN) PN1, PN3, PN5, and PN7. Spontaneous activity was evaluated with the open field test in adulthood. The cerebellum was evaluated for different parameters: microglial and Purkinje cell densities, oxidative stress levels, and tumor necrosis factor alpha (TNF-α) mRNA expression. Our results show that administration of LPS did not result in altered spontaneous activity in adult animals. Our data also indicate increased oxidative stress in the cerebellum, as evidenced by an increase in superoxide fluorescence by dihydroethidium (DHE) indicator. Stereological analyses indicated increased microglial density in the cerebellum that was not accompanied by Purkinje cell loss or altered TNF-α expression in adult animals. Interestingly, Purkinje cells ectopically positioned in the granular and molecular layers of the cerebellum were observed in animals of the LPS group. Our data suggest that neonatal LPS exposure causes persistent cellular and molecular changes to the cerebellum, indicating the susceptibility of this region to systemic inflammatory insults in infancy. Further investigation of the consequences of these changes and the development of strategies to avoid those should be subject of future studies.

## Introduction

Systemic inflammation in the premature neonate is associated with increased risk of adverse neurologic sequelae (Patra et al., 2017). Survivors of preterm birth may present long-term neurodevelopmental deficits, many of which are related to cerebellar injury, including impaired motor functions and also cognitive and behavioral dysfunction (Biran et al., 2012). The cerebellum is particularly vulnerable to insults since it is not fully developed until after birth in both humans and rodents (Biran et al., 2012). Understanding the pathophysiological mechanisms of brain injury caused by systemic inflammatory states in perinatal animals and its long-term consequences is of great relevance to neuroscience and might also have clinical relevance.

Administration of inflammatory agents is widely used in rodents to model prenatal, early, and late postnatal inflammation and generate changes that persist throughout life (Dean et al., 2015). Lipopolysaccharide (LPS) is an endotoxin released from Gram-negative bacteria that binds primarily to toll-like receptors (TLR)-4, leading to increased cytokine and chemokine expression in serum and brain (Buttini and Boddeke, 1995; Erickson and Banks, 2011). Acute LPS-induced inflammation in adult and aged rats causes blood–brain barrier disruption, oxidative stress, inflammation, microglial activation, neuronal loss, and white matter damage in different brain regions, including the cerebellum (Buttini and Boddeke, 1995; Qin et al., 2007; Banks et al., 2015; Pinato et al., 2015; Aslankoc et al., 2018; Garcia et al., 2018).

Early LPS exposure at various neurodevelopmental stages triggers a similar release of inflammatory cytokines (Romero et al., 2010; Fan et al., 2011; Falck et al., 2018) and causes sustained microglial activation and neuronal cell loss in brain areas such as the hippocampus, cerebral cortex, substantia nigra, ventral tegmental area, and thalamus (Ulmer et al., 2002; Fan et al., 2011; Wang et al., 2013; Zhu et al., 2014) and alters the levels of neurotransmitters in adult animals (Romero et al., 2010; Kubesova et al., 2015). Although a wide range of brain structures can be affected by perinatal LPS-induced inflammation, less is known with regard to effects over the cerebellum and related behavioral consequences. Of the few reports on the subject, we learn that early LPS administration in the perinatal period acutely decreases the cerebellar area (Cardoso et al., 2015a) and alters Purkinje cell development (Wallace et al., 2010; Xu et al., 2013; Hoffman et al., 2016; Zhang et al., 2018). Given the cerebellar vulnerability during the perinatal period and the already known influence of this structure to different motor, cognitive, and behavioral functions, the aim of this study was to investigate the long-term consequences of an early life inflammatory insult to the cerebellum of adult rats.

## Materials and Methods

All experimental procedures were approved by the Ethics Committee on Animal Research of Federal University of São Paulo (CEUA UNIFESP n: 9781020317). Animals were maintained in polypropylene cages under standard pathogen-free conditions in a 12-h light–dark cycle (06:00–18:00), under constant room temperature at 22 ± 2°C, with rat chow pellets and tap water provided *ad libitum*.

### Experimental Design

Wistar rats were mated at UNIFESP. The day of birth was defined as postnatal day 0 (PN0). The size of the litters was restricted to eight male offspring to minimize the effect of litter size on body weight. Male neonatal rats were randomly allocated to either LPS, sham, or naïve groups. LPS rats received intraperitoneal injections of LPS (1 mg/kg, *E*scherichia *coli*, 0111: B4; Sigma L-2630) on four alternate days from birth (PN1, PN3, PN5, and PN7) (Silva et al., 2019). Injections were applied at the same day period (7:00–10:00 a.m.). Sham animals received equal volume of sterile 0.9% saline injections. Age-matched rats that did not receive any injections were used as naïve controls. Animals were daily monitored during 15 days for abnormalities including sickness behavior (e.g., pale skin, lethargy, weight changes) and death. Rats were housed with their dams until PN 21, at which point they were weaned and divided into their adult groups. After that, rats were left undisturbed, except for weight monitoring. Starting at PN84, rats were subject to behavioral testing and were euthanized for tissue evaluation (PN89). The weight of the animals was assessed in such a way as to minimize effects of maternal care during the treatment period and at PN21, PN45, and PN89.

### Open Field Test

At first, we aimed to evaluate locomotor coordination and balance using the rotarod test in adult animals. However, subgroups of our tested rats (even naïve) were not able to stay in the rotating bean after PN80. We believe that this difficulty was due to size and body weight of the animals at that moment, and therefore, this particular test was dismissed from the protocol. Alternatively, the locomotor activity of rats was evaluated using the open field test.

The open field apparatus consisted of a circular wood arena measuring 97.5 cm in diameter, 34 cm high, under artificial light (40 lux) in a dedicated room. At PN84, each rat was gently placed in the center of the arena and allowed to explore it for 10 min. The locomotor activity was detected using an automated tracking system (EthoVision, Noldus Information Technologies, Netherlands) connected to a video camera positioned overhead. Using that automated tracking system, the arena was divided in a central zone (30 cm in diameter) and a peripheral zone. The number of entries and the time spent in each zone (center and periphery) were calculated. The total distance (cm) traveled, the mean velocity (cm per second), and time spent frozen (time that the animal remained immobile) were also calculated. All tests were performed in the afternoon (3:00–5:00 p.m.). At the end of each trial, the arena was wiped clean with 5% ethanol solution. For this analysis, we used *n* = 9 for the naïve group, *n* = 8 for the sham group, and *n* = 11 for the LPS group. Results were statistically analyzed using one-way ANOVA and are presented as mean ± SEM.

### Tissue Analysis

Rats were euthanized for tissue evaluation at PN89. For histological and immunohistochemical analysis, deeply anesthetized rats were perfused via the ascending aorta with phosphate buffer (PB) followed by 4% paraformaldehyde in PB. Brains were removed, postfixed in the indicated fixative for at least 24 h, and cryoprotected in a sucrose solution. Forty-micrometer-thick sagittal sections of the cerebellum were cut using a cryostat. For tumor necrosis factor alpha (TNF-α) mRNA measurement, a subset of rats was decapitated and the brains were quickly removed from the skull. The cerebellum was dissected on ice, snap frozen, and stored in a −80°C freezer.

### Immunohistochemistry for Microglial and Purkinje Cells

The ionized calcium-binding adaptor molecule 1 (Iba-1) was used for microglial staining and calbindin was used for Purkinje cell staining. Briefly, fixed sections of the cerebellum were washed in 0.1 M PB and incubated in 3% H_2_O_2_ for 20 min for autofluorescence quenching, followed by PB rinsing. Non-specific staining was blocked by incubating the tissue for 30 min in 3% bovine fetal serum, 0.2% Triton X-100 in PB. Sections were next incubated in primary antibodies (goat anti-Iba1, 1:500; ab5076 Abcam and mouse anti-Calbindin-D-28K, 1:3500; C9848 clone CB-955, Sigma-Aldrich) diluted in blocking solution, rotating overnight at room temperature. Sections were then washed in PB and incubated with secondary fluorescent antibody Alexa 488 donkey anti-goat and Alexa 546 goat anti-mouse, respectively (1:500; Invitrogen), rotating at room temperature for 120 min. The tissue was next rinsed in PB baths and incubated in DAPI (4′,6-diamidino-2-phenylindole; 1:10,000; D9564 Life Technologies) in PB for 10 min, to allow nuclear visualization. Brain sections were then mounted onto glass slides and coverslipped with Fluoromount (Southern Biotech).

The number of cells labeled for Iba1 and calbindin was estimated on the same animals using the optical dissector method (West et al., 1991). Analysis was performed using a microscope (Nikon Eclipse 80i) with a motorized stage connected to a computer running the Stereo Investigator software (MBF Bioscience). The cerebellum was examined in six sections for each rat, each section 240 μm apart, between Bregma −9.12 and −12.00 mm. For microglial cells, the right cerebellar cortex was considered for counting, and for Purkinje cells, the Purkinje cell layers presented in this same region were considered. These regions of interest were delineated using 10× objective lenses and cell counting was performed with 40× objective lenses. Based on a preliminary population estimate, a counting frame of 70 × 70 μm was distributed in a randomly positioned lattice of 600 × 600 μm for Iba1 counting and a counting frame of 50 × 50 μm was distributed in a randomly positioned lattice of 400 × 400 μm for calbindin counting. Section thickness after tissue processing varied between 23 and 32 μm. For this analysis, we used *n* = 5 for the naïve group, *n* = 4 for the sham group, and *n* = 6 for the LPS group. Results were statistically analyzed using one-way ANOVA and are presented as density of cells/mm^3^ ± SEM. In addition, we also performed morphological analysis on a sample of microglial cells to evaluate changes on the resting or activated state (see Supplementary Material for further detail). We investigated the association between the different immunohistochemical data by means of the Pearson Correlation test.

### Superoxide Anion Detection

Superoxide anion (O_2_^–^) was detected with the oxidative fluorescent probe dihydroethidium (DHE) (Sigma D7008), which reacts with superoxide anion. Briefly, fixed sections were washed in 0.1 M PB and incubated in 5 μM DHE in PB for 15 min. The tissue was next rinsed in PB baths and counterstained with DAPI (1:10,000; D9564 Life Technologies) in PB for 10 min. Sections were then mounted onto glass slides and coverslipped with Fluoromount (Southern Biotech). Five different fields of three cerebellum sections (15 total images) per animal were captured with 20× objective lenses using a confocal microscopy (Zeiss LSM 780). DHE fluorescence was detected with 510–560-nm excitation and 590-nm emission filters. DAPI fluorescence was detected with 330–380-nm excitation and 420-nm emission filters. Quantitative analysis of the fluorescence intensities was performed using ImageJ (Schneider et al., 2012). Briefly, the “Measure” tool was used for the assessment of fluorescence of the photomicrographs taken in different planes (z-plot). In this section, the “Stacks” and “Plot-2-axis” tools were employed. The data was allocated in the “IntDen” column of the “Results” table. The raw integrated density (IntDen) is the sum of all the pixel intensities in the ROI. That is what the 3D objects counter plugin uses for quantification. For regular 2D analysis (Analyze, Set Measurements) ImageJ reports both the “raw integrated density” and the “integrated density” which is the product of the area and mean intensity. Therefore, the IntDen values of all fluorescence channels relative to the IntDen value fluorescence of the cell nuclei provided by DAPI give us the pixelation value of each fluorophore used. The same animals used for the immunohistochemistry studies were also used for DHE analysis, *n* = 5 for the naïve group, *n* = 4 for the sham group, and *n* = 6 for the LPS group. Results were statistically analyzed using one-way ANOVA, and fluorescence values are expressed as the DHE/DAPI ratio in arbitrary units (A.U.). The association between immunohistochemical data and superoxide anion detection (DHE/DAPI fluorescence ratio) was analyzed by means of the Pearson correlation test.

### Semi-Quantitative PCR Analysis for TNF-α

Fresh tissue was homogenized in TRIzol Reagent (Invitrogen, United States) (1 mL). The purified RNA (as TRIzol protocol) was resuspended in 22 μL of sterile water at 60°C, and 2 μL of this solution was used for quantification in a spectrophotometer (ND-1000 NanoDrop Technologies, Wilmington, NC, United States) for subsequent cDNA synthesis. After quantification, 2 μg of total RNA was used for synthesis of the complementary DNA strand. To this was added 1 μL of RNA Oligo (dT) 15 primer (Promega, Madison, WI, United States) and RNase-free H_2_O to complete the 10-μL volume. This mixture was incubated for 5 min at 70°C followed by 5 min at 4°C. Then, 0.5 μL of RNasin Ribonuclease Inhibitor (Promega), 1 μL of 10 mM deoxynucleotide triphosphate (dNTP Promega), 1.0 μL of ImProm-II Reverse Transcription System (Promega), 4 μL of enzyme buffer, 1.5 μL of 25 mM MgCl_2_, and RNase-free H_2_O for a final volume of 20 μL were added. The conditions used for amplification in a thermocycler (Eppendorf) were 25°C for 5 min, 42°C for 60 min, and 70°C for 15 min. After synthesis, the cDNA samples were stored at −20°C. To verify the efficiency of real-time PCR, a reaction of conventional PCR was performed for amplification of TNF-α. The qPCR reaction was performed using Fast SYBR Green qPCR Master Mix (Applied Biosystems, United States) in a thermocycler 7500 Fast Real-Time PCR System (Applied Biosystems). The primer sequences for TNF-α used in this study were 5′-ACCTTATCTACTCCCAGGTTCT-3′ (forward) and 5′-GTCAGCCGATTTGCCATTTC-3′ (reverse). For each reaction, 20 ng of cDNA was added to 5 μL of SYBR Green, 0.4 μL of each primer (forward and reverse, 10 μM), and RNase-free H_2_O to 10 μL final volume. All reactions were performed in triplicate. The expression of TNF-α was normalized by the mean of hypoxanthine-guanine phosphoribosyltransferase (Hprt) and β-actin (Actb) gene expression and calculated using the 2^–ΔΔCt^ method. For this analysis, we used *n* = 3 for the naïve group, *n* = 3 for the sham group, and *n* = 4 for the LPS group. Results were statistically analyzed using one-way ANOVA.

## Results

### LPS Administration in the Early Neonatal Period Increased Mortality Rate and Led to Acute Weight Loss

**FIGURE 1:**
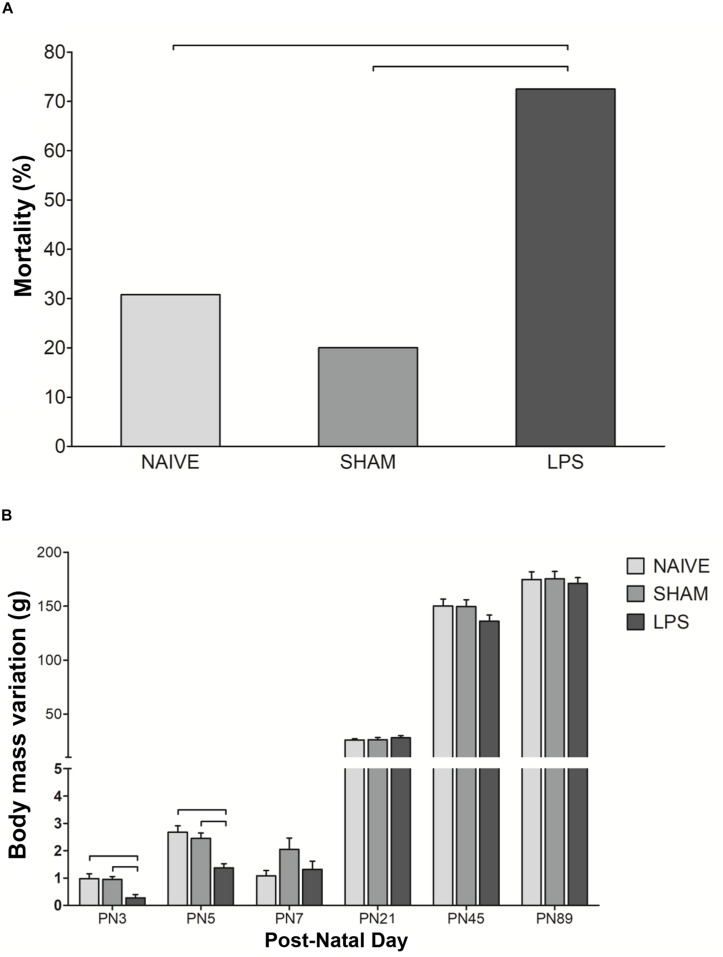
Mortality rate and weight change after neonatal LPS-induced inflammation. **(A)** Mortality rate in the LPS group was higher than in control groups. Values: percentage, χ^2^(2) = 13.006; bars: *p* < 0.01. **(B)** Neonatal LPS administration caused transient body weight change. Values: mean ± SEM, ANOVA, Tukey *post hoc* test, bars: *p* < 0.01 vs control groups on PN3 and PN5.

To assess the impact of LPS exposure in the first postnatal week, we initially monitored animals for abnormalities including sickness behavior. LPS pups presented pale skin and lethargy during treatment, indicating its toxicity and suggesting the occurrence of the inflammatory process. The mortality rate of pups in the LPS group (29/40, 73%) was higher than in the other groups [naïve: 4/13, 31%; sham: 2/10, 20%. Pearson χ^2^(2) = 13.006; *p* < 0.001; Bonferroni-adjusted alpha levels of 0.0083]. All deaths occurred between PN1 and PN12. In addition, body weight gain was acutely decreased by LPS, more expressively in PN3 and PN5 (*p* < 0.01) (Figure 1). At later time points, body weight oscillations were similar between the LPS group and untreated rats (Figure 1).

### Long-Term Locomotor Activity Was Not Altered by Neonatal Peripheral LPS Challenge

To verify the long-term influence of neonatal LPS administration in the general activity, rats were submitted to the open field test at PN84. No significant differences were detected between groups in any analyzed parameter (Table 1). All rats traveled around the arena the same distance [*F*(2,25) = 1.352; *p* = 0.277] with similar walking velocity [*F*(2,25) = 1.291; *p* = 0.293]. All animals exhibited comparable levels of anxiety as indicated by the absence of significant differences between groups in the number of central crossings [*F*(2,25) = 2.458; *p* = 0.094], time spent in central zone [F(2,25) = 1.382; *p* = 0.270], and motionless time [F(2,25) = 1.253; *p* = 0.303].

**TABLE 1:** Open field test in adult animals after neonatal peripheral LPS challenge (PN84).

Exploratory behavior and locomotor activity	Naïve (*n* = 9)	Sham (*n* = 8)	LPS (*n* = 11)
Travelled distance (cm)	5110 ± 110.2	4657.1 ± 240.2	4872 ± 108.7
Velocity (cm/s)	8.6 ± 0.2	7.8 ± 0.3	8.1 ± 0.3
Total time in central zone (s)	29.3 ± 5.9	40.7 ± 6.8	42.7 ± 5.8
Immobility (s)	541.2 ± 5.1	547.6 ± 7.2	533.5 ± 6.3

*Values: mean ± SEM. ANOVA, *p* > 0.05.*

### Long-Lasting Oxidative Stress Is Induced by Early Neonatal LPS Administration

**FIGURE 2:**
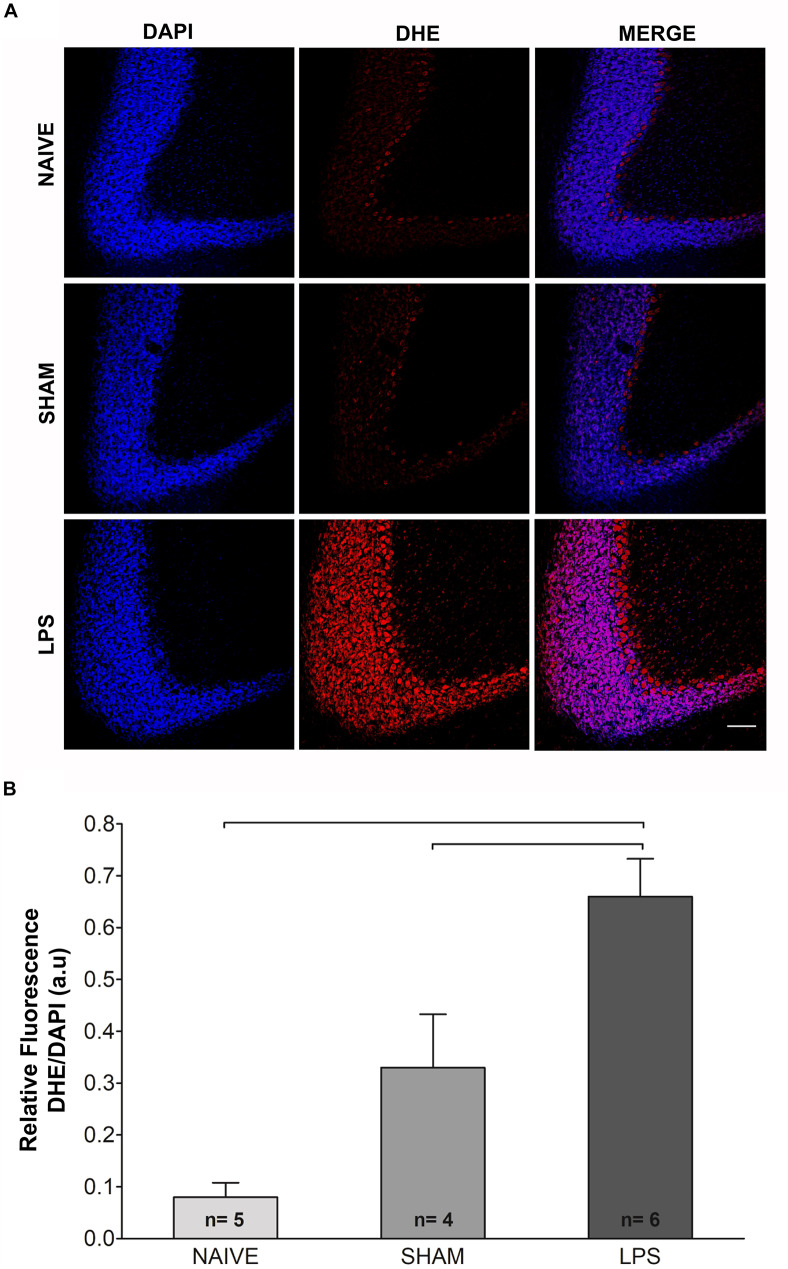
Photomicrographs and fluorescence ratio of reactive oxygen species in the cerebellum of adult rats (PN89) after early neonatal LPS induced inflammation. **(A)** Increased signal fluorescence of DHE staining in the LPS group as compared to control groups. Calibration bar: 200 μm. **(B)** Quantification of the DHE/DAPI fluorescence ratio shows marked increase in the LPS group. Values: mean ± SEM; ANOVA, Tukey *post hoc* test, bars: *p* < 0.001 vs control groups. DHE, dihydroethidium.

An indirect measurement of superoxide anion was performed through DHE reaction 3 months after neonatal LPS challenge (Figure 2). Adult animals in the LPS group presented higher DHE/DAPI fluorescence ratio as compared to naïve and sham groups [*F*(2,11) = 22.100; *p* < 0.001] (Figure 2).

### Neonatal Inflammation Increases Microglial Density in the Cerebellum of Adult Rats

**FIGURE 3:**
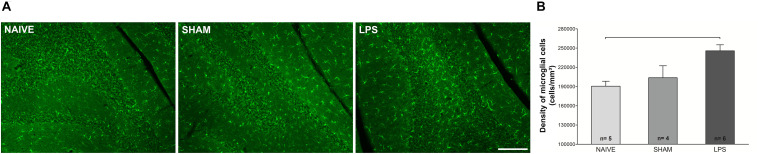
Photomicrographs and microglial densities in the cerebellum of adult rats (PN89) after early neonatal LPS-induced inflammation. **(A)** Representative immunofluorescence of microglial cells in the three animal groups. Calibration bar: 200 μm. **(B)** The density of Iba1 + cells increased in the LPS group. Values: mean ± SEM. ANOVA, Tukey *post hoc* test, bar: *p* < 0.01 vs naïve.

The morphology and density of Iba1-immunoreactive microglia was estimated in the cerebellum 3 months after neonatal LPS challenge. Using the dissector method in design-based stereology, increased microglial density was observed in the LPS group compared to naïve group [*F*(2,12) = 6.927; *p* < 0.01] (Figure 3). In addition, the LPS group presented a trend of difference as compared to the sham group [*F*(2,12) = 6.927; *p* = 0.064]. There was no difference between naïve and sham control groups [*F*(2,12) = 6.927; *p* = 0.729]. Morphological analysis showed no overt changes in microglial cell populations, with most cells exhibiting signs of resting state in both LPS and control groups (see Supplementary Material). There was a positive correlation between the microglial density and the DHE/DAPI fluorescence ratio (*r* = 0.589, *p* = 0.021).

### LPS Administration in the Early Neonatal Period Does Not Alter Neuronal Density but Changes Purkinje Cell Location in the Cerebellum

**FIGURE 4:**
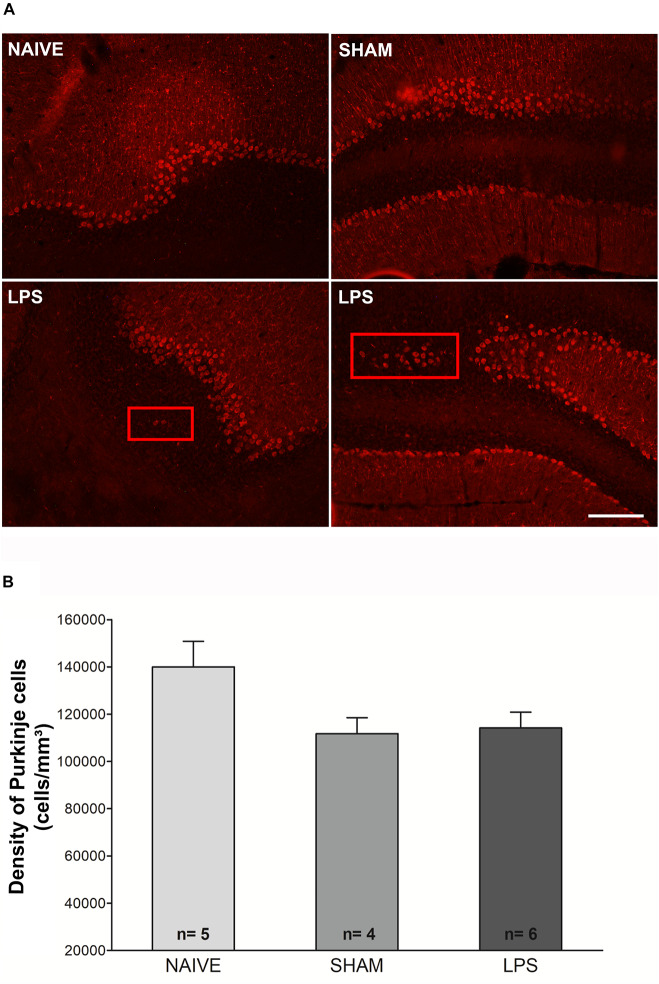
Photomicrographs and Purkinje cell densities in the cerebellum of adult rats (PN89) after early neonatal LPS induced inflammation. **(A)** Representative immunofluorescence of calbindin + cells in the three animal groups. Note the presence of Purkinje cells positioned outside the Purkinje cell layer (red rectangles) in LPS animals. This was not observed in control groups. Calibration bar: 200 μm. **(B)** There was no significant difference in Purkinje cell density among groups. Values: mean ± SEM. ANOVA, *p* > 0.05.

The density of calbindin-immunoreactive Purkinje cells was estimated in the cerebellum 3 months after neonatal LPS challenge. Statistical comparisons indicate a tendency to difference of Purkinje cell density between naïve, sham, or LPS groups [*F*(2,12) = 3.427; *p* = 0.066] (Figure 4). There was also a trend toward a negative correlation between the density of Purkinje cells and the DHE/DAPI fluorescence ratio (*r* = −0.486, *p* = 0.066). We found no correlation between the density of Purkinje and microglial cells (*r* = −0.263, *p* = 0.344). Interestingly, in LPS animals, it was observed that occasional Purkinje cells were located outside the Purkinje cell layer. The ectopic cells were present in the molecular layer or in the granular cell layer in all animals of the LPS group. In contrast, no animal of the naïve or sham control groups presented ectopic Purkinje cells (Figure 4).

### TNF-α mRNA Concentration Was Not Altered in Adult Rats Submitted to Systemic Inflammation as Neonates

**FIGURE 5:**
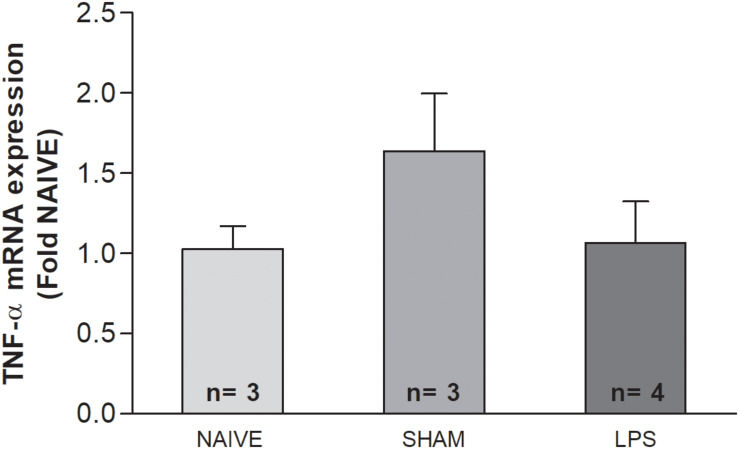
The mRNA level of TNF-α in the cerebellum of adult rats (PN89) after early neonatal LPS induced inflammation. The TNF-α mRNA concentration was not altered in adult rats submitted to systemic inflammation as neonates. Values: bars represent the ΔCt values (± SEM) of TNF-α mRNA expression normalized to the mean of Hprt and Actb. ANOVA, *p* > 0.05.

The concentration of TNF-α mRNA was investigated through qPCR in the cerebellum of adult rats, 3 months after LPS administration as neonates. There were no significant differences in TNF-α mRNA concentration between the LPS group and untreated rats in the cerebellum [*F*(2,7) = 1.502; *p* = 0.273] (Figure 5).

## Discussion

The current study investigated the long-term consequences of neonatal induced inflammation by systemic LPS injection on cerebellum. We demonstrated that the cerebellum of LPS-challenged animals present increased oxidative stress and increased microglial cell density in adulthood. In addition, the cerebellum of those animals also presents ectopic Purkinje cells. Thus, our data indicate that early-life systemic inflammation may induce permanent changes in the cerebellum of adult animals.

It has been demonstrated that during the first week of life, the immune system is qualitatively and quantitatively immature as compared to that of adulthood (Georgountzou and Papadopoulos, 2017). The differences between neonatal and adult immunity have been reported in both primates (humans) and rodents. The mechanisms that provide immunity in the neonates are functionally immature, characterized by the insufficient B-cell function, antibody production, and T-cell response (Adkins et al., 2004). The maturation process varies individually and is dependent on different molecules, cells, biochemical pathways, signaling, and antigen exposure. This immaturity of the immune system early in life imposes higher morbidity and mortality rates from infectious events.

Today, there is a great diversity of perinatal inflammation/infection models available in the literature (Dean et al., 2015). Among the many existing studies in this field, relatively few reports selectively investigated the cerebellum changes following LPS-induced perinatal inflammation in rodents (Wallace et al., 2010; Xu et al., 2013; Cardoso et al., 2015a; Hoffman et al., 2016). Importantly, previous studies focusing at the cerebellum used protocols of maternal infection rather than postnatal inflammation (Wallace et al., 2010; Xu et al., 2013) or applied low LPS doses (Xu et al., 2013; Hoffman et al., 2016) or did not evaluate long-term sequels (Cardoso et al., 2015a).

Here, we injected a relatively high dose of LPS (1 mg/kg) on alternate days from PN1 to PN7, a period when newborn rodents exhibit cerebellar cortex development consistent with the human fetus at 24–32 weeks (Biran et al., 2012). This injection protocol was designed to reduce the desensitization effect of multiple doses of LPS (modified from Stolp et al., 2005) and was previously established by our group (Silva et al., 2019). Here, we observed an acute loss in body weight following LPS administration indicating sickness behavior in neonates. Animals eventually recovered the body weight, but nevertheless, the neonatal mortality was high in LPS animals. Although we cannot exclude that handling neonates for experimental procedures and cross-fostering for litter size adjustment might have had some effect on mortality rate, we believe that the high mortality was a direct effect of the neonatal inflammatory response triggered by LPS injections (Zhao et al., 2008).

At PN84, almost 3 months after the neonatal inflammatory challenge, adult animals showed normal locomotor activity and anxiety levels in the open field test. This is consistent with previous reports that analyzed anxiety-like behavior (Silva et al., 2019) and with a recent study that demonstrated that various aspects of exploration and locomotion, social behavior, and cognition remained unaffected in adult rodents following repetitive high doses of LPS injections in neonates (Vojtechova et al., 2018). In addition, that study only reported inadequate emotional reactions to novel and stressful situations (Vojtechova et al., 2018). Similarly, previous studies using various LPS administration protocols to induce inflammation in the perinatal period also reported unaltered functional activity in adulthood (Roberson et al., 2006; Fan et al., 2011; Stolp et al., 2011; Rousset et al., 2013; Zhu et al., 2014). One particular study using LPS injection into the uterus reported impaired motor coordination on the rotarod in adulthood (mostly with faster rotation), but not on general locomotion (Wallace et al., 2010). As already stated, in the present study the rotarod test could not satisfactorily be deployed after PN80 to allow further investigation. Other reports showing altered motor activity performed related tests at earlier periods (<2 months) following LPS challenge (Girard et al., 2009; Stigger et al., 2013; Xu et al., 2013) or following a second LPS administration in adulthood (Bernardi et al., 2014; Tenk et al., 2008). Together, those data indicate that neonatal LPS challenge may lead to transitory motor dysfunction that might reach functional recovery in adulthood (Fan et al., 2011; Rousset et al., 2013).

The impact of neonatal inflammation can occur in several moments (short and long term) following LPS insult. It has been reported that repetitive high doses (6 mg/kg) of LPS in neonates trigger transient shrinkage of cerebellar layers and transient neuronal loss in the cerebellum. The authors also observed cerebellar hypoplasia and neuronal atrophy coincident with reduced cerebellar myelination in the week following repeated neonatal LPS administration (Cardoso et al., 2015a). Consistent with this finding, in maternal immune challenge models, the Purkinje cell layer was found to be less developed, and the soma size and cell density of Purkinje cells were decreased at a later time point (Wallace et al., 2010; Zhang et al., 2018). Similarly, abnormal Purkinje cell arborization also was observed following LPS-induced inflammation in specific time points after birth (Hoffman et al., 2016) and in primary cerebellar cultures (Xu et al., 2013). In the present study, we observed a tendency to a decrease of the Purkinje cell density (*p* = 0.06) 3 months after neonatal LPS challenge. It is possible that this tendency could reach statistical difference by increasing the number of animals. In spite of this lack of change in the cell density, we observed that some Purkinje cells were located outside the Purkinje cell layer in LPS-treated animals.

Reelin has a crucial prenatal role on the laminar organization of the cortex, cerebellum, and hippocampus, so that neurons recognize their proper location and orientation at the end of their migration pathway (Tissir and Goffinet, 2003). In cerebellum, reelin is secreted by cerebellar granule cells and recognized by specific binding sites expressed by Purkinje cells (Tissir and Goffinet, 2003; Leto et al., 2016; Beckinghausen and Sillitoe, 2019). In knock-in mice lacking the reelin C-terminal region, some Purkinje cells form clusters and are ectopically located in selected regions of the deep cerebellar mass (Nakamura et al., 2016). It is interesting to note that prenatal LPS infection can cause reductions in postnatal expression of reelin in the hippocampus (Harvey and Boksa, 2012; Nouel et al., 2012; Depino, 2015). In addition to transient reelin deficits, prenatal LPS challenge induces early and long-lasting pyramidal cell disarray in the hippocampus. Interestingly, prenatal treatment with antioxidants was efficient in preventing those disorders (Novais et al., 2013). A report on the reelin expression in the cerebellum of LPS-challenged rats as neonates showed non-significant decreases in reelin gene expression in the cerebellum of young female rats (Xu et al., 2013). Further studies are needed to elucidate if ectopic Purkinje cells found in the present study are related to a reelin deficiency triggered by neonatal LPS-induced inflammation.

Indirect assessment of superoxide anion detection revealed that LPS-induced inflammation at the neonatal period also leads to a persistent increase in cerebellar oxidative stress at long term. Similar results were also observed in the hippocampus, but were reverted by the blockade of P2X7 receptor (P2X7R), a purinergic ATP-binding receptor (Silva et al., 2019). In different animal models, the presence of oxidative stress in the cerebellum influences the vulnerability of Purkinje cells to damage and may cause problems in the cerebellar neurodevelopment (Kern and Jones, 2006; Imosemi, 2013; Xu et al., 2013). The cerebellum is highly vulnerable to oxidative stress due to its low content of antioxidants as compared to other brain tissues (Chaudhary and Parvez, 2012). Oxidative insults in this region also result in rapid changes in glial cells (Imosemi, 2013), in addition to reductions of important cerebellar proteins, such as presynaptic proteins, neurofilaments, and proteins involved in GABAergic neurotransmission (Benagiano et al., 2007; Lopez et al., 2009). As aforementioned, prenatal treatment with antioxidants prevented pyramidal cell disarray in the hippocampus (Novais et al., 2013), and treatment with P2X7R antagonist reverted hippocampal oxidative stress (Silva et al., 2019) of prenatal LPS challenged animals.

Concomitant with high levels of oxidative stress, adult animals subject to LPS in the neonatal period also presented higher density of microglia in the cerebellum when compared to control groups. This is in agreement with a persistent increase in microglial density verified in the hippocampus of adult animals subject to systemic neonatal LPS injections (Sominsky et al., 2012). In addition, microglial activation was also verified in other brain regions, such as substantia nigra, striatum, hippocampus, cerebral cortex, and thalamus, following intracerebral injection of LPS in neonatal rats (Fan et al., 2011; Wang et al., 2013; Zhu et al., 2014). Our results expand these findings to the cerebellum, confirming that the microglial population in this region also is increased in adulthood in animals exposed to systemic neonatal inflammation.

It is important to highlight that both *in vitro* and *in vivo* studies showed that cerebellar microglia may behave differently from those in the thalamus, neocortex, and hippocampus (Tay et al., 2017; Stowell et al., 2018). Cerebellar microglia are less densely distributed than their cortical counterparts, are less ramified, make dynamic contacts with Purkinje neurons, and present different morphological and dynamic profiles (Stowell et al., 2018). During early postnatal life, cerebellar microglia are intimately involved in the programmed death of developing Purkinje cells, mainly through production of superoxide ions (Marín-Teva et al., 2004; Cardoso et al., 2015b). In the present study, all histological and immunohistochemical analyses were performed on the same animals. Correlation analyses between these parameters showed a positive correlation between microglial density and oxidative stress. Moreover, there was a tendency toward a negative correlation between oxidative stress and the density of Purkinje cells. These findings and the abovementioned published reports allow us to hypothesize that alterations in cerebellar microglial physiology may sustain increased oxidative stress. The potential contribution of these changes to Purkinje cell mislocation in the cerebellum of adult animals remains to be investigated.

Given the long-lasting increase of microglial density and oxidative stress level in adulthood, we also evaluated TNF-α expression in the long term after neonatal inflammation. This cytokine was chosen because other authors observed prolonged expression of TNF-α (10 months) concomitant with microglial activation in brain structures after LPS challenge in adults (Qin et al., 2007; Bossù et al., 2012). We found no alteration in TNF-α mRNA expression in the cerebellum of adult animals. Corroborating our results, Walker et al. (2010) reported that neonatal LPS challenge did not alter TNF-α level in the hippocampus of adult animals. In that study, increased levels of TNF-α and IL-1β were only found in animals undergoing a second stressor stimulus in adulthood (Brochu et al., 2011). On the other hand, due to the nature of our experimental design, we cannot exclude an early transient increase in brain TNF-α expression, or even early transient increase of other inflammatory cytokines, such as IL-1β, IL-10, and CCL2 as reported by different studies (Stolp et al., 2005; Brochu et al., 2011; Stigger et al., 2013; Falck et al., 2018; Zhang et al., 2018). In such cases, early transient increased levels of inflammatory cytokines after a neonatal LPS challenge may contribute to microglial and oxidative changes that could be observed at long term in the cerebellum, as shown here.

Interestingly, increased expression of IL-1β or IL-6, but not TNF-α, concomitant with microglial activation, was verified in different brain regions following intracerebral injection of LPS in neonatal rats (Fan et al., 2011; Wang et al., 2013). Moreover, prolonged expression of IL-2, IL-6, and TNF-α also was verified in blood serum of adult animals exposed to prenatal LPS (Romero et al., 2010). Therefore, we cannot exclude that other cytokines could be chronically overexpressed in the cerebellum of our animals. In this regard, and as already pointed out, studies making use of high doses of LPS in adult animals showed prolonged expression of TNF-α in the brain, even at 10 months after insult (Qin et al., 2007; Bossù et al., 2012). Specifically, chronic TNF-α increase, together with IL-18 increase, was observed in the hippocampus, frontal cortex, and cerebellum (Bossù et al., 2012). It is possible that maintenance of the neuroinflammatory response for such a long period was due to the older age of animals at the time of LPS administration, or related to differences in the LPS protocol used to induce perinatal inflammation, which greatly differ from the systemic neonatal challenge used in the present study (Erickson and Banks, 2011; Lopes, 2016). Future studies are required to confirm chronic expression of cytokines in the cerebellum, which is out of the scope of the present study.

## Conclusion

Our data suggest that neonatal LPS exposure causes persistent alterations to the cerebellum, including changes in the microglial population, oxidative stress, and altered positioning of Purkinje cells, thus indicating the susceptibility of this region to systemic inflammatory insults. Further studies of the consequences of these changes and development of strategies to avoid them are still necessary.

## Data Availability Statement

The datasets used and/or analyzed during the current study are available from the corresponding author on request.

## Ethics Statement

The animal study was reviewed and approved by the Ethics Committee on Animal Research of Universidade Federal de São Paulo (CEUA n: 9781020317).

## Author Contributions

JP planned the project design, conducted experiments, analyzed data, and helped with the manuscript. MF provided intellectual support and ideas for experimental procedures and design, and helped with data analysis and manuscript writing. CS helped with experimental procedures and sample processing. DR helped with behavioral tests. MC contributed with DHE staining and analyses. AM and TN contributed with qPCR procedures and analyses. AL helped plan the project design. LM supervised the project and provided intellectual support for experimental procedures, data analysis, and manuscript writing. All authors read and approved the final manuscript.

## Conflict of Interest

The authors declare that the research was conducted in the absence of any commercial or financial relationships that could be construed as a potential conflict of interest.

## Abbreviations

Actb: β-actin
CEUA UNIFESP: Ethics Committee on Animal Research of Federal University of São Paulo
cDNA: complementary DNA
DAPI: 4 ′, 6-diamidino-2-phenylindole
DHE: dihydroethidium
dNTP: deoxynucleotide triphosphate
Hprt: hypoxanthine-guanine phosphoribosyltransferase
Iba-1: ionized calcium-binding adaptor molecule 1
LPS: lipopolysaccharide
O_2_^–^: superoxide anion
PB: phosphate buffer
PCR: polymerase chain reaction
PN: postnatal days
P2X7R: receptor P2X7
RNA: ribonucleic acid
TLR: toll-like receptor
TNF- α: tumor necrosis factor alpha.
