# Supplementary material for: Lipopolysaccharide-Induced Systemic Inflammation in the Neonatal Period Increases Microglial Density and Oxidative Stress in the Cerebellum of Adult Rats
Source: Front Cell Neurosci. 2020 Jun 3;14:142. doi: 10.3389/fncel.2020.00142 (PMC7283979; doi:10.3389/fncel.2020.00142)
Supplement: Supplementary file 1 [file Data_Sheet_1.docx]

**SUPPLEMENTARY MATERIAL**

1. **Materials and Methods**

**Morphological Analysis of Microglia**

Immunostaining to identify microglia was carried out on tissue sections as previously described in the manuscript. Microglia morphology was analyzed to identify changes on the resting or activated state, such as alterations on cell body and cell ramification after LPS injection. Photomicrographs of microglia at 40X magnification (Nikon Eclipse 80i) from naïve, sham and LPS groups were used. All photomicrographs used for analysis were obtained from a single focal plane, in which microglia and cell processes are randomly oriented.

We used ImageJ software (National Institute of Health, https://imagej.nih.gov/ij/34) and appropriate plugins (filter, unsharp mask, close and FracLac) to convert all photomicrographs to binary, skeletonized and outline images to analyze cell morphology. In addition, cell somas were manually counted in each photomicrograph. Figure S1A illustrates the workflow process to convert an entire original photomicrograph to a plugin tagged image (original, binary, skeletonize and outline). Cropped photomicrographs are shown to better visualize microglia morphology detail; however, data were collected from uncropped images. We summarized the number of process endpoints and length from the Analyze Skeleton plugin (<http://imagej.net/AnalyzeSkeleton31>) data output and normalized all data by the number of microglia cell somas in each image to calculate the number of microglia endpoints/cell and microglia process length/cell. To compare microglia complexity, we used FracLac plugin for ImageJ (<https://imagej.nih.gov/ij/plugins/fraclac/FLHelp/Introduction.htm>), which quantifies each cell’s contour bounded by the endpoints and process lengths, calculating the fractal dimension. In addition, we evaluated the span ratios as indicators of cell shape, and density as indicator of cell size.

In Table S1, we summarize skeleton (endpoints and process length) and fractal analysis measures (density, fractal dimension and span ratio) in terms of measure, unit, sampling and interpretation.

**Table S1.** Summary of microglia morphology measures.

| **Microglia morphology** | **Measure** | **Unit** | **Sampling** | **Interpretation** |  |
| --- | --- | --- | --- | --- | --- |
| Endpoints | Summed | µm | 2 photomicrographs/animal | Cell ramification |  |
| Process length | Summed | #/cell | 2 photomicrographs/animal | Cell ramification |  |
| Fractal Dimension | Regression slope | D_B_ | 4 cells/animal | Cell complexity |  |
| Span Ratio | convex hull eclipse longest length convex hull eclipse longest width | ratio | 4 cells/animal | Cell shape |  |
| Density | # of pixels within cell outline area of convex hull | # of pixels area | 4 cells/animal | Cell size |  |

1. **Results**

**Microglial morphology remains unchanged in the cerebellum after LPS administration in the early neonatal period**

Examples of microglia (original, binary, skeletonized and outlined) in each group are shown in Figure S1A. The morphology of Iba1 immunoreactive microglia was estimated in cerebellum three months after neonatal LPS challenge. Several features were analyzed as endpoints, process length, cell size, cell complexity and cell shape. No difference was observed after statistic comparisons of the data from Analyze Skeleton and FracLac ImageJ plugins (Figure B-F).


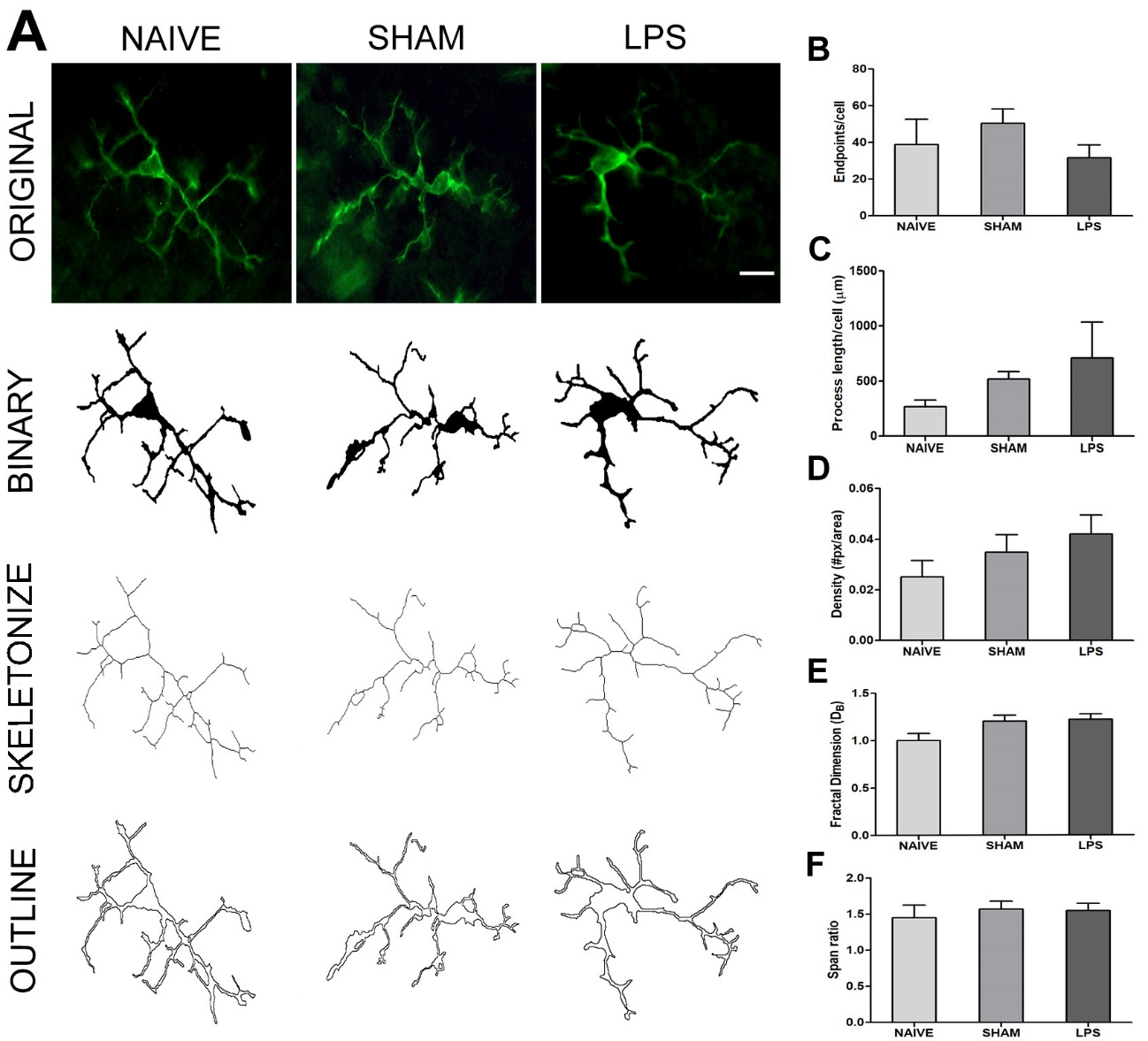


**Figure S1.** Skeleton and fractal analysis of microglia morphologies in Iba1 stained tissue. (A) The process to prepare photomicrographs for skeleton and fractal analysis. Original photomicrographs were subjected to a series of uniform ImageJ plugin protocols prior to conversion to binary images; binary images were then skeletonized or outlined. (Scale bar = 10 µm). (B-F) The skeletonized and outlined images were processed using the Analyze Skeleton or FracLac plugins to identify several morphological measures. Results were statistically analyzed using one-way Anova. No difference between groups was observed (p>0.05).
